# Cerebral Oxygen Saturation During Less‐Invasive Surfactant Administration Using a High‐Pressure CPAP Respiratory Support Delivery Room Protocol—A Cohort Study

**DOI:** 10.1111/apa.70591

**Published:** 2026-05-14

**Authors:** Jan Trieschmann, Anika Verhoef, Angela Kribs, Katrin Mehler, Benjamin Kuehne, André Oberthuer

**Affiliations:** ^1^ Division of Neonatology, Department of Pediatrics, Faculty of Medicine and University Hospital Cologne University of Cologne Cologne Germany

**Keywords:** bronchopulmonary dysplasia, lung recruitment, preterm infant, respiratory distress syndrome, surfactant

## Abstract

**Aim:**

To describe changes in peripheral oxygen saturation (SpO_2_) and regional cerebral oxygenation (rcSO_2_) during less invasive surfactant administration (LISA) in a high‐level continuous positive airway pressure (CPAP) respiratory support delivery room protocol in very low birth weight infants (VLBW).

**Methods:**

This is a secondary analysis of data from the randomized‐controlled Extrauterine Placental Transfusion in Resuscitation of VLBW infants (EXPLAIN) trial. In total, 38 patients were included. The pressure and changes in heart rate, SpO_2_ and rcSO_2_ were assessed 10 min before until 10 min after the LISA procedure. Basic outcome data for the patient collective were collected.

**Results:**

Mean gestational age was 27 + 6 weeks (±15 days), and mean birth weight was 995.8 g (±298.8). All infants were eligible for LISA. During LISA, heart rate, SpO_2_ and rcSO_2_ decreased significantly (mean change 32.4 (± 27.5) bpm, −14.5 (± 10.7) % and −11.9 (± 9.1) %, respectively; all *p* < 0.001). Moreover, two infants developed a pneumothorax and three infants required endotracheal intubation within 72 h after birth.

**Conclusions:**

With the approach of a high‐level CPAP setting, we observed transient decreases in heart rate and oxygenation levels during LISA. The magnitude of these decreases was within the expected or potentially favourable range compared with prior studies.

**Trial Registration:**

ClinicalTrials.gov identifier:NCT03916159

## Summary

This study evaluated high‐level continuous positive airway pressure (CPAP) during less invasive surfactant administration using a Benveniste valve as a CPAP generator in very low birth weight infants. Significant transient decreases in heart rate, peripheral oxygen saturation and regional cerebral oxygenation were observed but remained within expected or favourable ranges compared with previous studies. Also low rates of intubation and pneumothoraxes indicate the safety of this approach.

**Abbreviations**
BPD: bronchopulmonary dysplasia
CPAP: continuous positive airway pressure
FRC: functional residual capacity
LISA: less invasive surfactant application
MAP: mean airway pressure
PEEP: positive end expiratory pressure
rcSO_2_
: regional cerebral oxygen saturation
RDS: respiratory distress syndrome
SpO_2_
: peripheral oxygen saturation
VLBW: very low birth weight

## 1 Introduction

Respiratory distress syndrome (RDS) remains a major cause of morbidity in very low birth weight (VLBW) infants. Less invasive surfactant administration (LISA) in spontaneous breathing infants on continuous positive airway pressure (CPAP) support has been adopted as first‐line therapy for RDS, improving short‐ and long‐term outcomes [1]. Surfactant administration, irrespective of the technique, represents a period of physiological instability. Several studies have reported transient drops in peripheral oxygen saturation (SpO_2_) and cerebral regional oxygen saturation (rcSO_2_) during less invasive surfactant administration [2, 3, 4], raising questions about how best to optimize lung volume and respiratory drive before and during LISA in order to minimize fluctuations in systemic and cerebral oxygen delivery that could affect the long‐term neurodevelopment [5, 6, 7].

During initial lung recruitment and LISA, current guidelines recommend the use of CPAP with pressure levels at levels of initially 6 cm H_2_O [1]. However, experimental data suggest that a high‐pressure approach with a gradual increase in pressure in the range of 7–25 cmH_2_O may lead to improved functional residual capacity (FRC) without an increase of adverse side effects [8, 9]. Preliminary small clinical studies have reported that a high‐level CPAP approach facilitates postnatal adaptation in VLBW infants [10, 11], but large trials are still ongoing [12]. To date, though, high‐level CPAP concepts have mainly been evaluated in the context of respiratory and cardiovascular stabilization during foetal to neonatal transition and the impact of high‐level CPAP on subsequent procedures such as LISA, particularly with respect to SpO_2_ and rcSO_2_ dynamics, has not been systematically studied.

This observational study therefore aimed to investigate the effects of a high‐level CPAP approach during LISA on SpO_2_ and rcSO_2_ in VLBW infants. The present study reports on prespecified secondary outcomes from the Extrauterine Placental Transfusion in Resuscitation of Very Low Birth Weight Infants (EXPLAIN) study.

## 2 Methods

### 2.1 Study Side and Recruitment

This study presents an analysis of prespecified secondary outcome data from infants enrolled in the EXPLAIN study, a single‐centre, randomized controlled trial conducted at the University Hospital of Cologne, Germany, between 2019 and 2021 [13]. Infants born by caesarean delivery with birth weight < 1500 g and gestational age > 23 + 6 weeks of gestation were eligible for the EXPLAIN trial. The exclusion criteria for the primary study included vaginal delivery; foetal or maternal risks; placental abruption or placenta previa with haemorrhage; placental anomalies; monochorionic multiples; and congenital anomalies after discharge. All study data were collected from maternal and infant medical records and managed using REDCap electronic data capture tools hosted at the University of Cologne. REDCap (Research Electronic Data Capture) is a secure, web‐based software platform designed to support data capture for research studies. This article follows the current Strengthening the reporting of observational studies in epidemiology (STROBE) reporting guidelines.

### 2.2 Procedures

After Caesarean delivery, infants were transferred to the resuscitation unit, where neonatal staff supported the infant's transition essentially as described by Mehler et al. [11]. CPAP was provided via face mask with a variable flow CPAP device (*Benveniste valve, Dameca, Copenhagen, Denmark*) [14, 15]. FiO_2_ was initially set in the range of 0.21–0.30 in infants less than 28 weeks' gestational age, and a gas flow of 14 L/min was used resulting in a pressure of approximately 8–10 cm H_2_O. Depending on the infants' breathing efforts, heart rate and SpO_2_, gas flow was increased every 30 s by 2 L/min to a maximum of 22 L/min, resulting in a maximum pressure of approximately 30 cmH_2_O [13]. Supplemental oxygen was adjusted according to SpO_2_ targets based on the reference ranges reported by Dawson et al. [16]. After the primary stabilization, the CPAP interface was switched from the face mask to a nasopharyngeal tube and when eupnoeic spontaneous breathing was established, the flow was reduced stepwise. Caffeine citrate was administered at a dose of 20 mg/kg body weight after establishing peripheral venous access [17]. The LISA procedure was performed on stable and spontaneous breathing infants when pre‐defined criteria were met (Silverman score > 5 or FiO_2_ > 0.3 or > 14 L of gas flow, corresponding to a pressure of approximately 12 cm H_2_O). Surfactant (*CUROSURF, Chiesi Pharmaceuticals Parma, Italy*) was applied with 200 mg/kg body weight by laryngoscopy with a specific catheter (*LISACath, Chiesi, Parma, Italy* or *surfcath, Vygon, Aachen, Germany*) directly into the trachea [11]. In case of instability FiO2 and flow were increased. Prior to the LISA procedure, all infants were manually held in a facilitated tucking position by a nurse for several minutes and were kept in this position during LISA. In addition, non‐nutritive sucking on a swab with 20% glucose was performed as a non‐pharmacological measure in some infants [18]. A few infants received 0.5–1.0 mg/kg of ketamine as an analgesic and sedative measure prior to the LISA procedure. The indication for ketamine was determined individually at the discretion of the attending neonatologist orientating at the COMFORTneo score [19].

### 2.3 Data Collection

SpO_2_ and heart rate were measured with a Masimo 7 SET pulse‐oximeter (*Masimo Radical, Masimo Corporation, Irvine, California, USA*). Cerebral oxygenation was measured by near‐infrared spectroscopy (*FORESIGHT, Casmed, Branford, Connecticut, USA*), with sensors placed on infants' foreheads after transferring to a resuscitation bed. Regional cerebral oxygen saturation (rcSO_2_) was recorded synchronously with cardiorespiratory parameters. These data and respiratory rate and airway pressure were recorded with the New Life Box ALD resuscitation monitor (*Advanced Life Diagnostics, Weener, Germany*) with 200 Hz. The data was analyzed in combination with a synchronized video of the procedure to detect relevant intervention. Every 30 s, the values were averaged over an interval of 10 s and checked for artefacts. The onset of the successful laryngoscopy for the surfactant administration via LISA was determined as time zero. Small for gestational age (SGA) was defined as birth weight below the 10th percentile. IVH was staged according to the criteria of Papile et al. [20]. Bronchopulmonary dysplasia was categorized according to the definition of Walsh et al. [21].

### 2.4 Statistical Analysis

Statistical analysis was performed using IBM SPSS Statistics for Macintosh, Version 30/31 (IBM Corp, New York, USA). Data are presented as mean and standard deviation (SD), as median and interquartile range (IQR), or absolute and relative frequencies. The changes of the parameters were compared to the baseline before the procedure. Because the data did not show a normal distribution, the Wilcoxon rank‐sum test was used for paired comparisons. A two‐tailed *p*‐value < 0.05 was considered statistically significant. No sample size calculation was performed because this study used data for predefined secondary outcome.

### 2.5 Ethics

The EXPLAIN study was approved by the local ethics committee of the medical faculty of the University of Cologne (18–232). Parents of all enrolled infants provided verbal and written informed consent prior to participation. The study was registered in clinicalTrials.gov (NCT03916159).

## 3 Results

**FIGURE 1:**
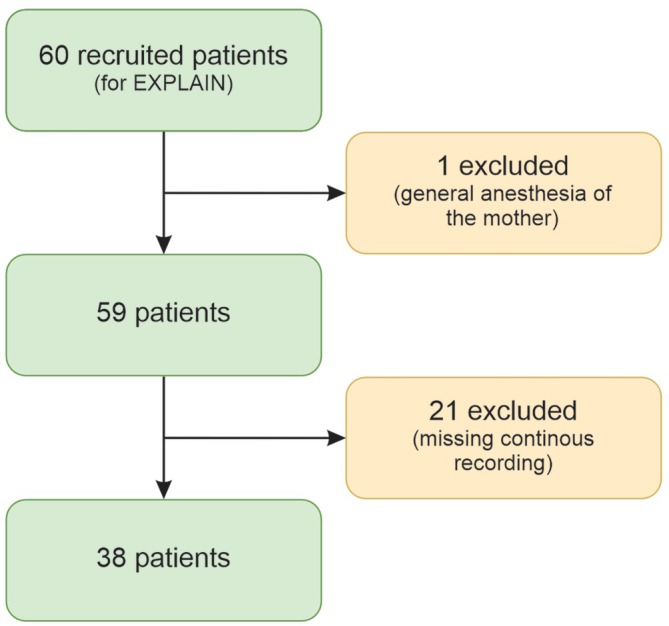
Flowchart patient recruitment.

A total of 38 infants from the original trial had complete recordings of cardiorespiratory function monitoring and were included in the analysis (Figure 1). Out of these 38 patients, 19 were female and 19 were male; the mean (± standard deviation [SD]) gestational age was 27 + 6 weeks (±15 days); the average weight was 995.8 (±298.8) *g*. Intravenous Caffeine was given within a mean of 18.8 (± 5.6) minutes after birth. LISA was performed at a mean of 41.8 (± 9.5) minutes after birth, with an average of 73.3 (± 25.4) seconds from the beginning of the laryngoscopy until the completed application.

**FIGURE 2:**
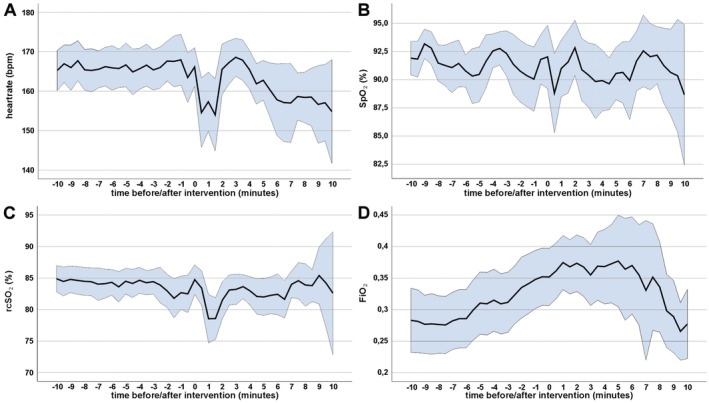
Data presented as means and 95% CIs. *X*‐axis: Minutes before/after the intervention. (A) Heart rate in beats per minute. (B) Peripheral oxygenation (SpO_2_) in % (C) Cerebral oxygen saturation (rcSO_2_) in % (D) FiO_2_.

Following LISA, significant changes were observed in all measured vital parameters. Heart rate decreased from baseline (mean over the 10 min prior to successful surfactant administration) of 166.2 (± 14.1) bpm to a mean minimum of 133.8 (± 28.5) bpm, corresponding to a mean reduction of 32.4 (± 27.5) bpm (*p* < 0.001). SpO_2_ declined from a mean of 91.4 (± 3.8) % at baseline to a nadir mean of 77.0 (± 11.3) % at the minimal value, representing a mean decrease of 14.5 (± 10.7) % (*p* < 0.001). Regional cerebral oxygen saturation (rcSO_2_) similarly decreased from a mean of 84.0 (± 5.3) % to 72.1 (± 10.1) %, with a mean decline of 11.9 (± 9.1) % (*p* < 0.001) (Figure 2).

In 25 infants (65.8%) LISA was successful on the first attempt. In 8 infants (21.0%) two attempts, and in 4 infants (10.5%), three attempts were necessary. Ketamine was administered in 4 infants (10.5%). In the delivery room, no intubation was necessary; within the first 72 h, three patients required invasive mechanical ventilation (7.9%). Pneumothoraxes occurred in two patients (5.3%) of whom only one required drainage (2.6%) with no pneumothoraxes in the first 24 h. Further basic clinical outcomes are shown in Table 1.

**TABLE 1:** Patient data and basic outcome.

Categories	
Gestational age, mean (± SD)	27 + 6 weeks (±15)
Sex	
Female, *n* (%)	19 (50%)
Male, *n* (%)	19 (50%)
Birth weight, mean (± SD)	995.8 g (±298.8)
Apgar	
1 min, median (IQR)	6 (5 to 7)
5 min, median (IQR)	8 (7 to 8)
10 min, median (IQR)	8 (8 to 9)
Intubation, *n* (%)	8 (21.1%)
Delivery room (%)	0 (0%)
Within first 72 h, (%)	3 (7.9%)
Survival, *n* (%)	37 (97.4%)
Pneumothoraxes, *n* (%)	2 (5.3%)
BPD	
Mild, *n* (%)	22 (57.9%)
Moderate, *n* (%)	1 (2.6%)
Severe, *n* (%)	0 (0%)
FIP, *n* (%)	6 (15.8%)
NEC, *n* (%)	2 (5.3%)
IVH	
I°, *n* (%)	7 (18.4%)
II°, *n* (%)	1 (2.6%)
III°, *n* (%)	1 (2.6%)
PVL, *n* (%)	1 (2.6%)
ROP (treatment necessary), *n* (%)	1 (2.6%)

Mean CPAP level measured between the Benveniste valve and the pharyngeal tube within 10 min prior to the surfactant administration until 10 min after the intervention was 23.7 (± 3.0) cmH_2_O. The mean FiO_2_ until the intervention was 30.4 (± 11.6)%, during the procedure, an increase of FiO_2_ was necessary with a mean maximal FiO_2_ of 42.1 (± 18.9)%. 10 min after the surfactant administration an average FiO_2_ of 27.7 (± 7.2)% was observed.

## 4 Discussion

The present study assessed the impact of LISA combined with non‐invasive high‐level CPAP support on systemic and cerebral oxygenation in VLBW infants. To our knowledge, this is the first study to specifically evaluate changes in peripheral oxygen saturation (SpO_2_) and cerebral oxygenation (rcSO_2_) in this setting. We observed significant, transient decreases in both parameters, with mean declines of 14.5% in SpO_2_ and 11.9% in rcSO_2_.

A high‐level CPAP strategy with early lung recruitment may improve respiratory stability by promoting functional residual capacity and maintaining alveolar recruitment during LISA. This aligns with evidence suggesting that higher or dynamic MAP strategies are not associated with lung injury when excessive inflation pressures are avoided. In animal models, it has been shown that high inflation pressure was more injurious than dynamic pressure up to 15 cm H_2_O [22]. Similarly, a cohort study reported lower delivery room intubation rates with a dynamic pressure approach compared with positive pressure ventilation using pressure levels of 6–8 cm H_2_O [23]. In a retrospective cohort study, Kanaan et al. also described improved outcomes after implementing a dynamic pressure with sustained inflation protocol [24]. Furthermore, positive pressure ventilation has been shown to generate higher tidal volumes than spontaneous breathing [25]. Although the pressure levels in these studies were lower than the high‐level CPAP approach applied here, the overall data supports the concept that limiting high inflation pressures while using adequate pressure to sustain recruitment may reduce lung injury.

Supporting this, all infants included in this study were eligible for LISA and none required delivery room intubation. Furthermore, the outcome in the studied cohort was favourable (Table 1). Despite the use of high‐level CPAP, pneumothorax occurred in 5.3% of infants but without any pneumothoraxes in the delivery room, which is within the expected range reported in the literature [26]. These findings suggest that combining LISA with lung recruitment using dynamic high‐level CPAP may represent a safe and effective approach for initial respiratory stabilization and surfactant administration in VLBW infants [11].

Comparative studies assessing cerebral oxygenation during LISA performed with CPAP levels of 5–8 cm H_2_0 have reported heterogeneous results. Bertini et al. described a pronounced decrease in rcSO_2_ of 30.1% (from 75.6% to 45.5%, *n* = 10) at pressure levels of 5–8 cm H_2_O, in a comparable cohort of preterm infants with mean gestational age of 30.2 weeks and a mean birth weight of 1399 g^4^. Hanke et al. reported that 30% of the infants experienced an rcSO_2_ drop of > 20%. However, no mean changes were reported here, limiting direct comparability [27]. In contrast, Li et al. observed a reduction in rcSO_2_ of only 4.9% (from 82.3% to 77.4%; *n* = 22) using binasal CPAP with pressure levels of 5–8 cm H_2_O in VLBW infants [3]. Notably, Li et al. quantified the change over the first 5 min after the procedure, whereas we assessed the maximal decrease over the full observation period.

In this context, the 11.9% decrease in rcSO_2_ observed in the present study lies within the mid‐range of values reported in the literature, suggesting that LISA under a high‐level CPAP approach is feasible without evidence of a greater decline in rcSO_2_ than reported in published cohorts. This is clinically relevant, as previous studies have demonstrated that decreases in rcSO_2_ are more frequently observed in patients with adverse outcomes, indicating a potential association between cerebral desaturation and impaired neurological or overall clinical prognosis [7]. Randomized controlled trials are warranted to study the clinical relevance of different CPAP levels during LISA.

With regard to sedation, there was no statistically significant difference in the magnitude of SpO_2_ or rcSO_2_ decline between infants receiving ketamine and those managed with non‐pharmacological measures. However, in several of those infants, oxygen saturation decreased prior to laryngoscopy because of transient hypo‐ or apnoeic episodes following drug administration. Given the small number of infants within expected or favourable ranges compared with previous studies receiving ketamine (*n* = 4), a robust analysis of post‐ketamine hypo/apnea was beyond the scope of this study and should be addressed in adequately powered future trials [28].

## 5 Strengths and Limitations

To our knowledge, this is the first study to report detailed systemic and cerebral oxygenation changes during LISA performed within a delivery room high‐level CPAP lung‐recruitment protocol in VLBW infants using continuously recorded physiologic parameters. Using this high‐level CPAP strategy, none of the 38 analyzed infants required delivery room intubation, which is notable given the degree of prematurity in this population.

However, there are relevant limitations: First, this study does not provide a direct within‐centre comparison but relates our local standard to data from other studies; therefore, differences in the clinical conditions (e.g., only Caesarean delivery), procedural details and equipment may have influenced the measured parameters. Second, the protocol did not standardize the operator performing LISA, and inter‐operator variability (including differences in experience) may have contributed to variability in the magnitude of measured parameter changes. Third, CPAP generation using the Benveniste valve may not be directly transferable to CPAP generation with T‐piece devices.

In addition, recordings of the LISA procedure were unavailable for 21 of 59 infants. Consequently, these infants were excluded from the present analysis because key procedural time points and measurement intervals could not be reliably ascertained retrospectively. Although baseline characteristics and neonatal outcomes of these infants appeared comparable, there is a potential for selection bias.

## 6 Conclusion

In summary, this study demonstrates that combining LISA with non‐invasive lung recruitment using a high‐level CPAP strategy is feasible and appears safe in VLBW infants. Although transient declines in heart rate, peripheral and cerebral oxygenation occurred during the procedure, the magnitude of these decreases was within the range of values reported in the literature. Importantly, the use of high‐level CPAP was associated with a low complication rate. No delivery room intubations were required, subsequent invasive ventilation during the hospital course was uncommon, and pneumothorax rates were at a level expected for the degree of prematurity of the infants. Randomized controlled trials are warranted to determine the impact of different CPAP level strategies during LISA on cerebral oxygenation, respiratory outcomes and long‐term neurodevelopment.

## Author Contributions

**Angela Kribs:** conceptualization, investigation, supervision, project administration, writing – review and editing. **Anika Verhoef:** investigation, data curation, writing – review and editing, formal analysis. **André Oberthuer:** conceptualization, investigation, supervision, writing – review and editing, project administration. **Katrin Mehler:** investigation, writing – review and editing. **Jan Trieschmann:** investigation, writing – review and editing, writing – original draft, formal analysis, visualization. **Benjamin Kuehne:** conceptualization, investigation, writing – review and editing, supervision, project administration, methodology, funding acquisition, data curation, formal analysis, visualization, writing – original draft.

## Funding

The study was supported in part by research grant no. 210‐01.01‐19 from the Marga and Walter Boll‐Stiftung (Kerpen, Germany) and by a research grant from the German Interdisciplinary Association for Intensive Care and Emergency Medicine (Deutsche Interdisziplinaere Vereinigung für Intensiv‐ und Notfallmedizin, Hamburg, Germany).

## Conflicts of Interest

The authors declare no conflicts of interest.

## Data Availability

The data that support the findings of this study are available from the corresponding author upon reasonable request.
